# Carbon and nitrogen metabolism affects kentucky bluegrass rhizome expansion

**DOI:** 10.1186/s12870-023-04230-x

**Published:** 2023-04-26

**Authors:** Fu Ran, Yajuan Yuan, Xiaoming Bai, Changning Li, Juanxia Li, Hui Chen

**Affiliations:** 1 College of Grassland Science, Gansu Agricultural University, Lanzhou, 730070 China; 2 Key Laboratory of Grassland Ecosystem, Gansu Agricultural University, Lanzhou, 730070 China

**Keywords:** Kentucky bluegrass, Metabolomics, Rhizome, Carbon, Nitrogen

## Abstract

**Background:**

Rhizome is vital for carbon and nitrogen metabolism of the whole plant. However, the effect of carbon and nitrogen in the rhizome on rhizome expansion remains unclear.

**Results:**

Three wild Kentucky bluegrass (*Poa pratensis* L.) germplasms with different rhizome expansion capacity (strong expansion capacity, ‘YZ’; medium expansion capacity, ‘WY’; and weak expansion capacity, ‘AD’) were planted in the field and the rhizomes number, tiller number, rhizome dry weight, physiological indicators and enzyme activity associated carbon and nitrogen metabolisms were measured. Liquid chromatography coupled to mass spectrometry (LC-MS) was utilized to analyze the metabolomic of the rhizomes. The results showed that the rhizome and tiller numbers of the YZ were 3.26 and 2.69-fold of that of the AD, respectively. The aboveground dry weight of the YZ was the greatest among all three germplasms. Contents of soluble sugar, starch, sucrose, NO_3_^−^-N, and free amino acid were significantly higher in rhizomes of the YZ than those of the WY and AD (*P* < 0.05). The activities of glutamine synthetase (GS), glutamate dehydrogenase (GDH) and sucrose phosphate synthase (SPS) of the YZ were the highest among all three germplasm, with values of 17.73 A·g^− 1^ h^− 1^, 5.96 µmol·g^− 1^ min^− 1^, and 11.35 mg·g^− 1^ h^− 1^, respectively. Metabolomics analyses revealed that a total of 28 differentially expressed metabolites (DEMs) were up-regulated, and 25 DEMs were down-regulated in both comparison groups (AD vs. YZ group and WY vs. YZ group). Kyoto Encyclopedia of Genes and Genomes (KEGG) pathway enrichment analysis demonstrated that metabolites related to histidine metabolism, tyrosine metabolism, tryptophan metabolism, and phenylalanine metabolism were associated with rhizomes carbon and nitrogen metabolism.

**Conclusions:**

Overall, the results suggest that soluble sugar, starch, sucrose, NO_3_^−^-N, and free amino acid in rhizome are important to and promote rhizome expansion in Kentucky bluegrass, while tryptamine, 3-methylhistidine, 3-indoleacetonitrile, indole, and histamine may be key metabolites in promoting carbon and nitrogen metabolism of rhizome.

**Supplementary Information:**

The online version contains supplementary material available at 10.1186/s12870-023-04230-x.

## Background

Rhizome is a modified, horizontal underground stem of plant. It is a vital organ for nutrient storage, ramet generation, information and material exchange between cloned ramets and vegetative propagation for the rhizomatous plants [1–3]. Rhizomes with strong expansion capacity can generate more ramets, obtain more nutrients from soil, and better resist or tolerate stresses [4–6], It has been shown that turfgrasses with rhizomes, such as Kentucky bluegrass (*Poa pratensis* L.), are easier to establish as a turf, have greater resistance to stress [7], and can rapidly activate rhizomes to produce shoots and form new plants in post-disturbance regeneration [8, 9]. Therefore, understanding the process of rhizome growth and development is important for turfgrass growth and turf establishment.

The development of rhizome of turfgrass is affected by various factors, including carbohydrate and nitrogen levels in plant, environment and phytohormones. Plant carbohydrates and nitrogen status affect plant overwintering, rhizome bud germination, and community reproduction [10, 11]. The structural features of rhizomes are similar to those of tillers [12]. It was found that, exogenous sucrose can induce longer and more robust rhizomes in perennial rice (*Oryza longistaminata*) [13]. Higher levels of non-fibrous carbohydrates in rhizome benefited the post-disturbance regeneration of plants [14]. In the mutants of wheat (*tin*) and rice (*moc 2*), reduction in sucrose content led to inhibition of tiller growth [15, 16]. Furthermore, sucrose in plant can act as signaling entities. Wang et al. [17] found that sucrose affected the bud growth via glycolysis and oxidative pentose phosphate pathway (OPPP). Downregulation of carbon and nitrogen metabolism causes inhibition of cytosolic glutamine synthetase and reduction of tiller number in the plant [18]. Similar to carbon, nitrogen status also affects the rhizome growth. It was found that sufficient nitrogen promoted rhizome growth and increased rhizome bud density in plants [12, 19]. New rhizomes grow from internodes and genes related to nitrogen metabolism are upregulated in post-drought recuperation [9]. In rice (*Oryza sativa*) branching, tiller number positively correlated with plant total nitrogen accumulation [20], and high amino acid content in leaves is beneficial for tiller formation and growth [21]. Furthermore, appropriate N addition promotes asexual reproduction in Kentucky bluegrass [22]. The balance of C assimilation and N uptake controls stem and root growth of Arabidopsis thaliana (*Arabidopsis*) [23]. These results indicate that the nitrogen and carbon metabolism is important for the development and growth of rhizomatous plants.

Kentucky bluegrass (*Poa pratensis* L.) is a perennial rhizomatous plant that is widely distributed in cool and humid regions of temperate zones and is an important plant species of grassland in such areas [24]. This plant is also extensively used in lawns, for soil and water conservation, and ecological restoration because of its robust rhizome and good adaptability to the environment [25, 26]. Nevertheless, China is rich in wild Kentucky bluegrass germplasm resources. Under long-term natural selection, wild Kentucky bluegrass germplasm resources are expected to harbor excellent trait characteristics to adapt to local climates. Metabolomics is widely applied for better understanding phenotypic variation [27, 28], investigating nutritive values [29], and stress resistance [30, 31]. Luo et al. [32] reported 19 significantly different metabolites causing flavor attributes of the two types of *P. sibiricum* rhizomes. Masson et al. [33] successfully utilized metabolomics to discriminate 22 *Iris* rhizomes, and tentatively identified 32 metabolite discriminating markers to distinguish origins of these plants. Kentucky bluegrass varies greatly in its rhizome expansion capacities [34]. However, the effect of carbon and nitrogen metabolism on such a difference is yet to be investigated.

The purposes of the present work were to, (i) investigate the relationship between carbon and nitrogen metabolism and Kentucky bluegrass rhizome expansion capacity; (ii) investigate the relationship between enzymes, metabolism pathways, and metabolites and Kentucky bluegrass rhizome extension.

## Results

### Rhizome number, tiller number and plant dry weight

**Fig. 1:**
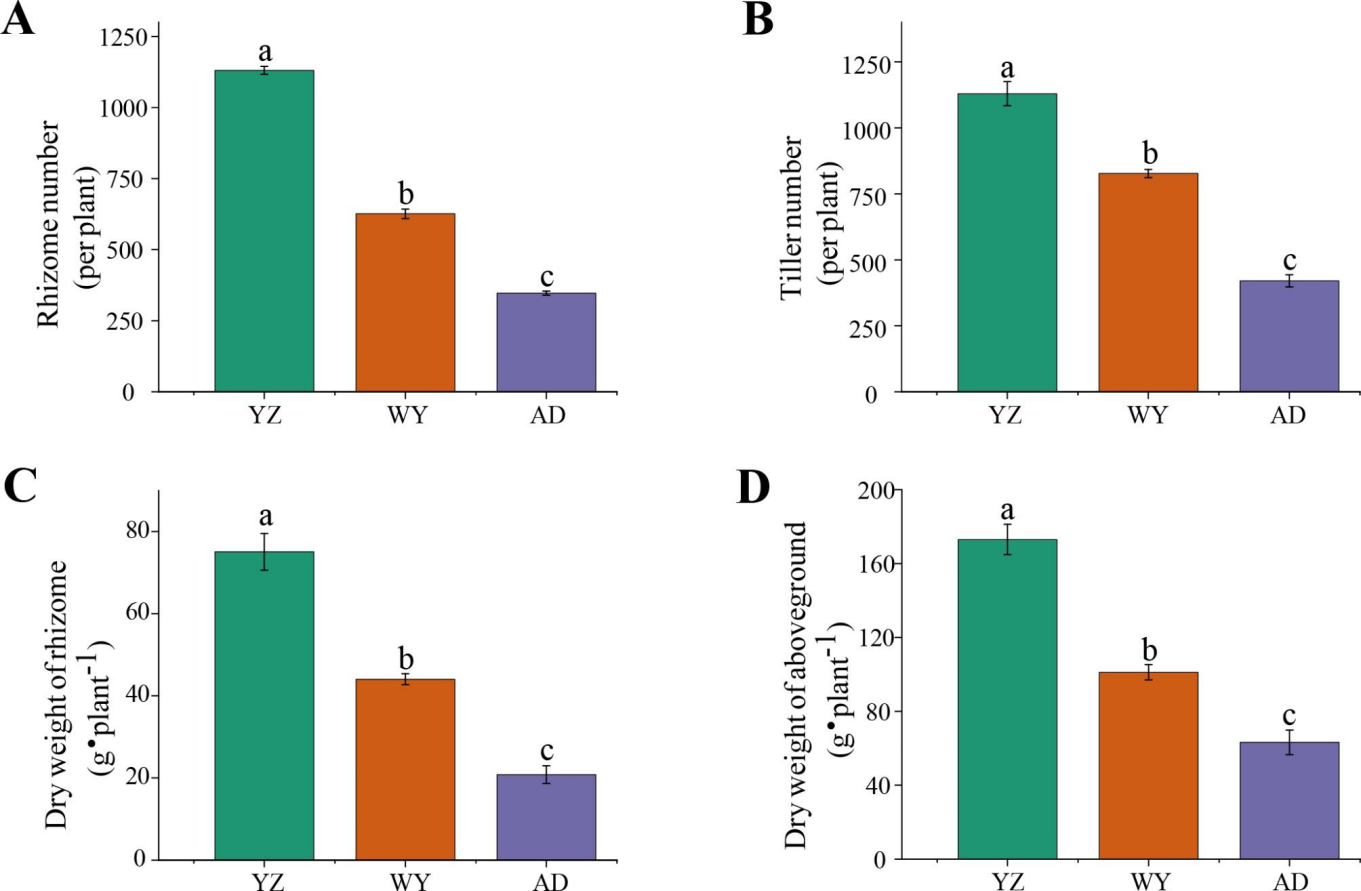
Rhizome number (**A**), tiller number (**B**), rhizome dry weight (**C**) and aboveground dry weight (**D**) of three Kentucky bluegrass germplasms. Different letters above the error bars of the same parameter indicate significant difference at *P* < 0.05

Rhizome number, tiller number, rhizome and aboveground dry weight are indicated in Fig. 1. Significant difference was found between all the three germplasms for all four parameters (*P* < 0.05). All four parameters ranked YZ > WY > AD. The rhizome number of the YZ were 1.81 and 3.26-fold of that of the WY and AD, respectively (Fig. 1A), while the tiller number were 1.36 and 2.69-fold of that of the WY and AD (Fig. 1B). Similarly, the rhizome dry weight of the YZ was 1.70 and 3.61-fold of that of the WY and AD, respectively (Fig. 1C), while the aboveground dry weight was 1.70 and 2.73-fold of that of the WY and AD (Fig. 1D).

### Rhizome C and N contents

**Fig. 2:**
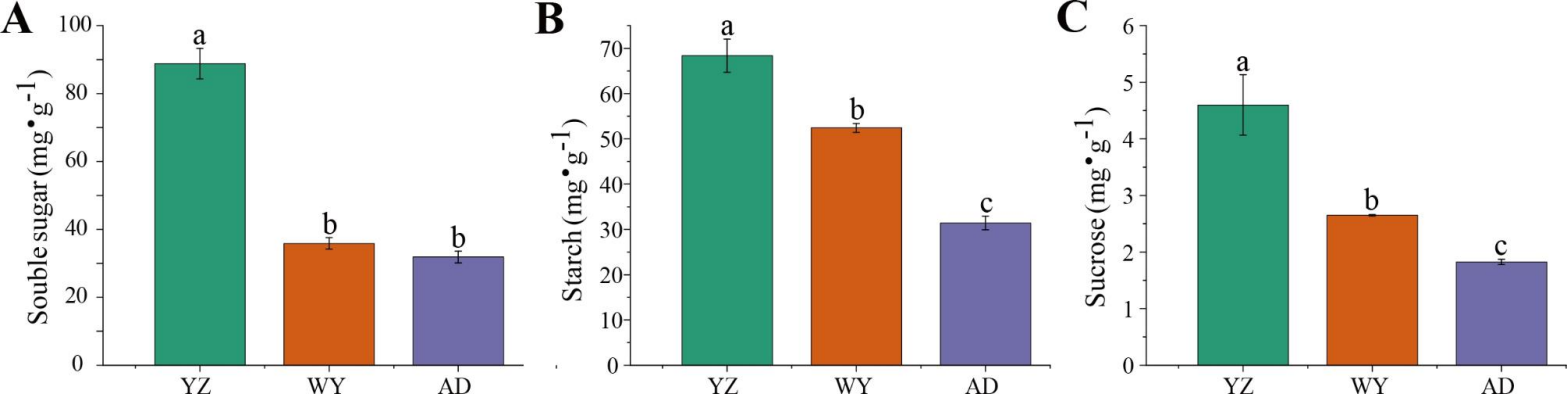
Soluble sugar content (**A**), starch content (**B**), and sucrose content (**C**) in the rhizomes of Kentucky bluegrass germplasms YZ, WY, and AD. Significant differences among materials are expressed by differing lowercase letters (*P* < 0.05)

The three Kentucky bluegrass germplasms differed markedly in their rhizome carbohydrate contents (Fig. 2). The soluble sugar, starch, and sucrose contents of the YZ were higher than those of the WY and AD (*P* < 0.05). The soluble sugar content of the YZ was 147.66% and 178.63% higher than that of the WY and AD, respectively, whereas the latter two had a similar content (Fig. 2A). Likewise, the sucrose content of the YZ was 73.6 and 153% higher than those WY and AD, respectively (Fig. 2C). The starch content of the YZ was 30.4% and 118% higher than those of the WY and AD, respectively.

**Fig. 3:**
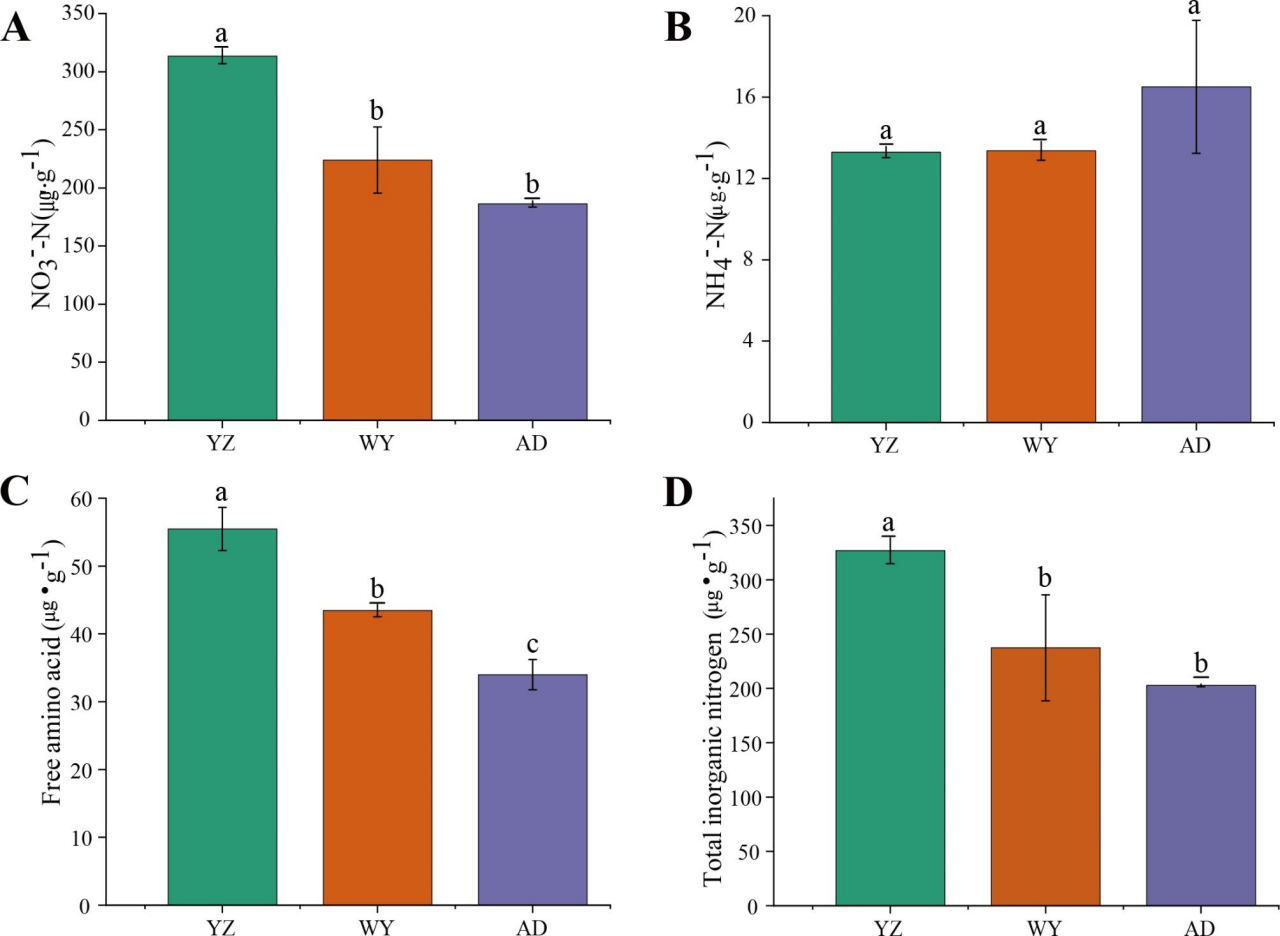
NO_3_^−^-N (**A**), NH_4_^+^-N (**B**), free amino acid (**C**), and Total inorganic nitrogen (**D**) contents in the rhizomes of Kentucky bluegrass germplasms YZ, WY, and AD. Significant differences among materials are expressed by differing lowercase letters (*P* < 0.05)

Contents of NO_3_^−^-N, NH_4_^+^-N, free amino acid content and total inorganic nitrogen are indicated in Fig. 3. The rhizome NO_3_^−^-N content of the YZ was significantly higher than those of the WY and AD (*P* < 0.05); however, no differences were found between values of the WY and AD (Fig. 3A). The NO_3_^−^-N content of the YZ was 40.0 and 68.3% greater than that of the WY and AD, respectively. Interestingly, no significant difference was found for NH_4_^+^-N contents between different germplasms, but that of the AD was higher than those of the YZ and WY (Fig. 3B). The free amino acid content was highest in the YZ, being 27. 8% and 63.3% higher than WY and AD, respectively (Fig. 3C). Total inorganic nitrogen of the YZ were higher than those of the WY and AD (*P* < 0.05) (Fig. 3D).

### Rhizome enzyme activity

Enzyme activities of the three Kentucky bluegrass germplasms are indicated in Table 1. The enzyme activities of different Kentucky bluegrass ranked YZ > WY > AD, except for those of SS, NI and AI, the YZ had significantly greater enzyme activities than AD or both WY and AD for the activities of GS, GDH and SPS(*P* < 0.05), while for SS, the activities ranked AD > WY > YZ (*P* < 0.05). The rhizome GS, GDH and SPS activities of the YZ were 25.9–92.4% greater than those of the WY and AD.

**Table 1:** Enzyme activities in rhizome of three Kentucky bluegrass germplasms with different expansion capacities

Enzyme	YZ	WY	AD
Glutamine synthetase (GS) (A·g^− 1^ h^− 1^)	17.7 ± 1.0a	10.2 ± 0.36b	9.47 ± 0.28b
Glutamate dehydrogenase (GDH) (µmol·g^− 1^ min^− 1^)	5.96 ± 0.06a	5.77 ± 0.16a	4.20 ± 0.21b
Glutamate synthase (GOGAT) (µmol·g^− 1^ h^− 1^)	2.93 ± 0.30a	2.93 ± 0.57a	2.20 ± 0.57a
Nitrate reductase (NR) (µg·g^− 1^ h^− 1^)	10.9 ± 1.53a	9.93 ± 1.41a	8.18 ± 0.13a
Sucrose synthase (SS) (mg·g^− 1^ h^− 1^)	5.38 ± 0.25c	8.23 ± 0.31b	10.3 ± 0.26a
Sucrose phosphate synthase (SPS) (mg·g^− 1^ h^− 1^)	11.3 ± 0.27a	8.41 ± 0.43b	6.86 ± 0.70b
Neutral invertase (NI) (mg·g^− 1^ h^− 1^)	4.81 ± 0.61a	5.27 ± 0.41a	4.26 ± 0.52a
Acid invertase (AI) (mg·g^− 1^ h^− 1^)	3.93 ± 0.15a	5.09 ± 1.24a	4.06 ± 0.44a

Different lowercase letters behind the value in the same row indicate significant different at *P* < 0.05

### Principal component analysis

Morphological and physiological indicators accounted for 81.2% of the total variation (Additional file 3: Fig. S1). Among these, rhizome number, tiller number, rhizome dry weight, and aboveground dry weight of morphological indicators showed a high positive contribution. SPS, soluble sugar, starch, and sucrose (carbon metabolism) showed a high positive contribution. NO_3_^−^-N, free amino acid, GDH, and GS (nitrogen metabolism) showed a high positive contribution.

### Metabolite profiling

The PLS-DA was used to analyze the metabolomic information of different rhizomes, and score plots exhibited significant separation. Both its R2Y and Q2Y were close to 1, with R2Y > Q2 (Additional file 4: Fig. S2); In the AD and YZ groups (R2Y = 0.99, Q2Y = 0.90), the first principal component (PC1) and second principal component (PC2) explained 30.2% and 16.0% of the total variance, respectively (Additional file 4: Fig. S2A). In the WY and YZ groups (R2Y = 0.99, Q2Y = 0.89), the PC1 and PC2 explained 27.3% and 23.29% of the total variance, respectively (Additional file 4: Fig. S2B).

**Fig. 4:**
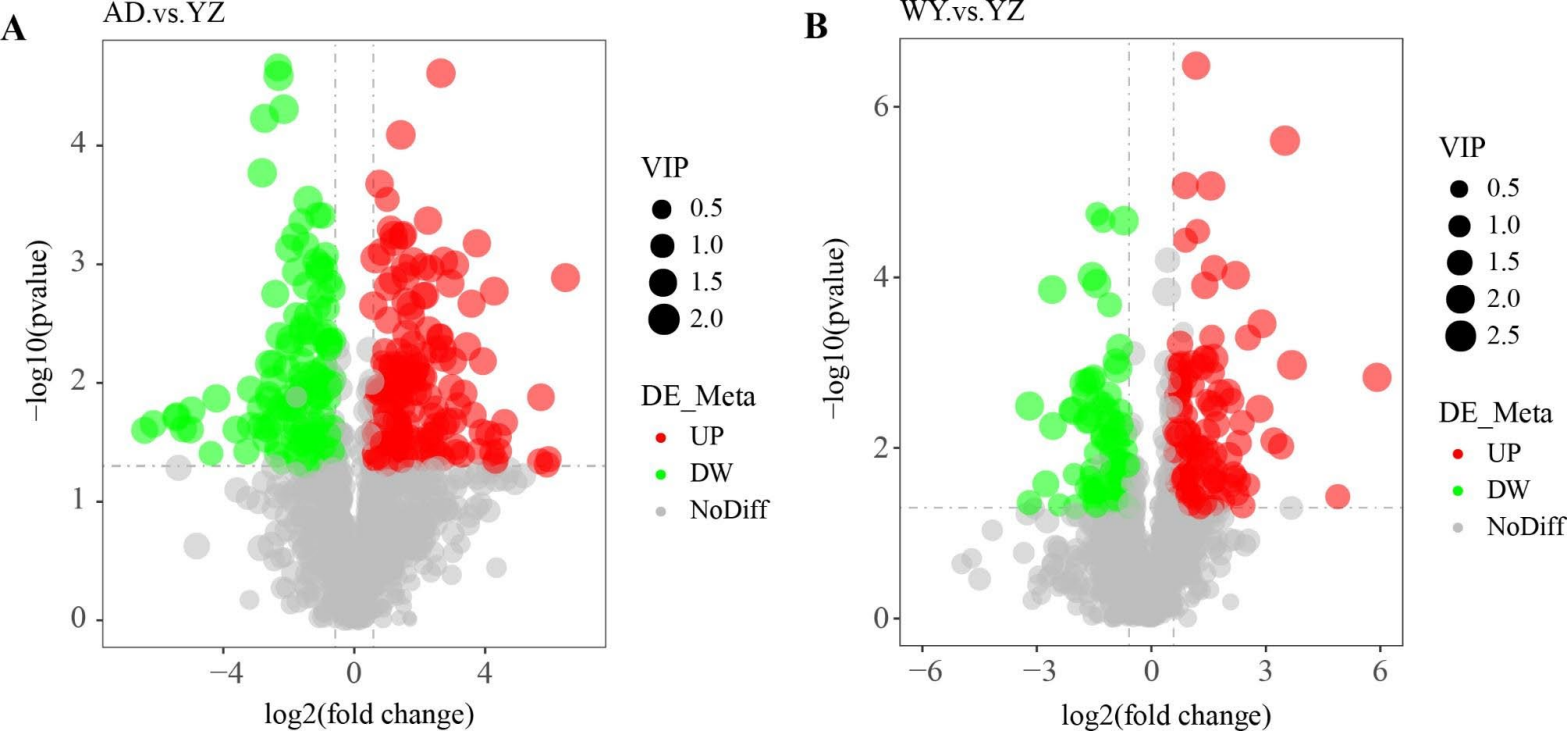
AD vs. YZ (**A**) and WY vs. YZ (**B**) volcano plot of differentially expressed metabolites (DEMs). Each dot represents a metabolite, and the size of the dot represents the VIP value. Red dots represent up-regulated metabolites, green dots represent down-regulated metabolites, and grey dots represent unchanged metabolites

A total of 321 differentially expressed metabolites (DEMs) between AD and YZ (188 up-regulated, 133 down-regulated; Fig. 4A), and 214 DEMs between WY and YZ (133 up-regulated, 81 down-regulated; Fig. 4B) were identified. Among these differential metabolites, 28 metabolites were simultaneously upward-regulated, and 25 metabolites were simultaneously downward-regulated in both AD vs. YZ group and WY vs. YZ group.

### Metabolic pathways related to carbon and nitrogen metabolism

**Fig. 5:**
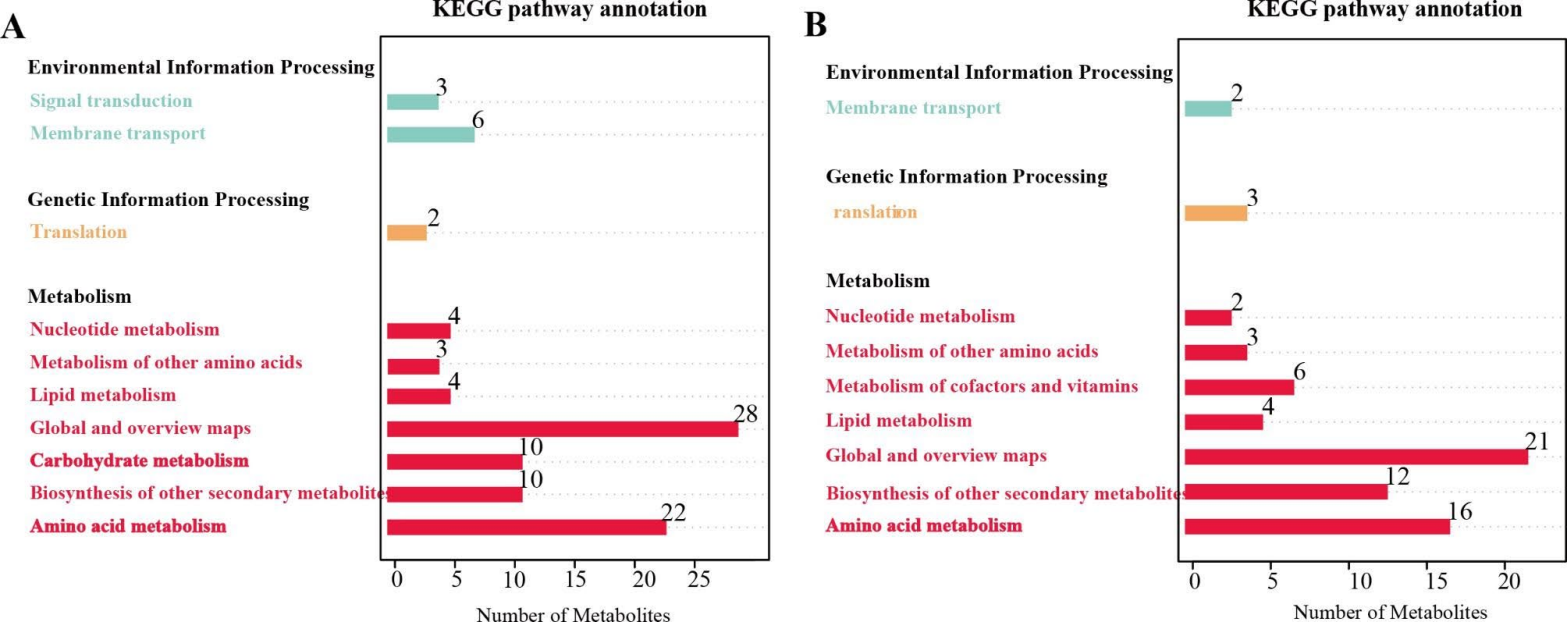
AD vs. YZ (**A**) and WY vs. YZ (**B**) differentially expressed metabolites (DEMs) in summary of KEGG annotation classification. Ordinate and abscissa represent the annotated KEGG pathway and number of metabolites, respectively

**Fig. 6:**
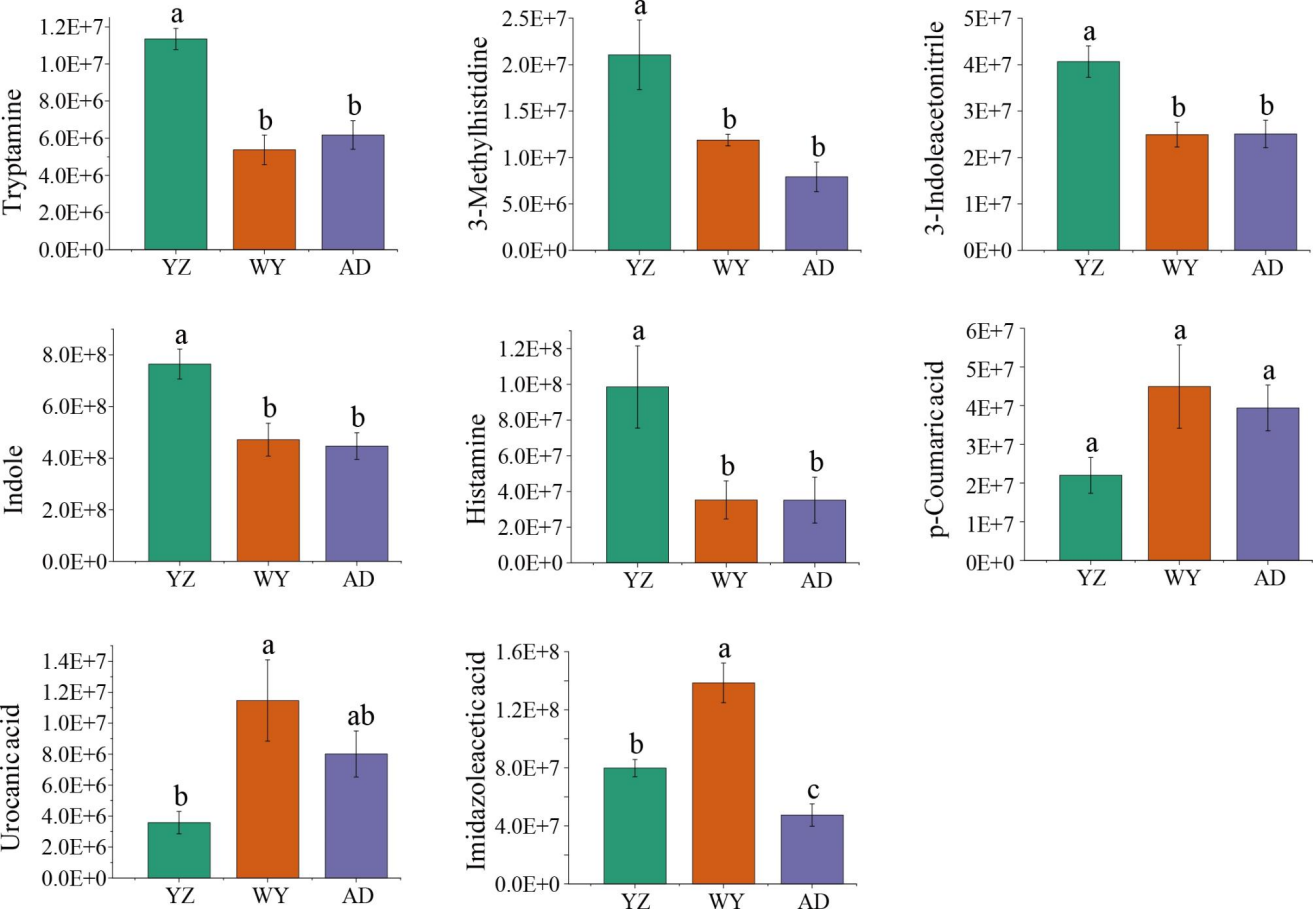
Content of key differentially expressed metabolites (DEMs). Their significant differences are indicated by differing lowercase letters (*P* < 0.05)

Based on Kyoto Encyclopedia of Genes and Genomes (KEGG, Fig. 5) and KEGG pathway database (Additional file 5: Fig. S3), DEMs associated with carbon and nitrogen metabolism were identified, including those in amino acids and their derivatives, carbohydrates, alcohol, tricarboxylic acids and derivatives, indoles and derivatives, phenols, and other compounds. In the AD vs. YZ group, 29 DEMs were associated with carbon and nitrogen metabolism (Fig. 5A; Table 2). Intriguingly, three metabolites were duplicated. In WY vs. YZ group, 16 DEMs were associated with nitrogen metabolism (Fig. 5B; Table 3). Finally, we obtained eight common DEMs in two comparison groups as shown in Fig. 6. Among these, tryptamine, 3-methylhistidine, 3-indoleacetonitrile, indole, and histamine were up-regulated and p-coumaric acid and urocanic acid were down-regulated in YZ compared to their levels in WY and AD. These DEMs were present for four metabolic pathways (histidine metabolism, tyrosine metabolism, tryptophan metabolism, and phenylalanine metabolism; Additional file 2: Table S2).

**Table 2:** Changes in differentially expressed metabolites in AD vs. YZ group rhizomes

Metabolite name	log_2_FC	*P*-value
Trans-Aconitic acid	-0.770140	0.027406
L-Serine	0.602874	0.025122
D-myo-Inositol 1,4-bisphosphate	-0.734220	0.048781
Stachyose	1.374013	0.028476
Citric acid	-0.878490	0.000845
Raffinose	1.653100	0.006071
Alpha-Ketoglutaric acid	-0.965880	0.000388
Dulcitol	1.294920	0.037261
D-Saccharic acid	-0.983440	0.007749
Inositol	2.649665	2.46E-05
Indole	-0.774200	0.004504
Hippuric acid	4.416546	0.037635
3-Indoleacetonitrile	-0.696610	0.010170
Salicylic acid	-0.877070	0.044835
L-Cystine	2.452311	0.027064
Tryptamine	-0.877420	0.004671
N6, N6, N6-Trimethyl-L-lysine	-0.786770	0.010650
L-Histidine	-0.907890	0.010943
Tyrosol	2.195468	0.030416
Picolinic acid	0.809101	0.013971
Homovanillic acid	1.369793	0.005398
Imidazoleacetic acid	-0.748610	0.030467
Betaine	0.877056	0.011661
5-Aminovaleric acid	0.744158	0.037212
p-Coumaric acid	0.840596	0.027549
Histamine	-1.486820	0.029002
3-Methylhistidine	-1.410390	0.002744
Urocanic acid	1.161847	0.044715
N-Acetyl-L-phenylalanine	1.741616	0.002749

**Table 3:** Changes in differentially expressed metabolites in WY vs. YZ group rhizomes

Metabolite name	log_2_FC	*P*-value
3-Methylhistidine	-0.826240	0.024257
Rosmarinic acid	-1.781110	0.001739
Urocanic acid	1.679588	0.024064
3-Indoleacetonitrile	-0.704990	0.006800
Indole	-0.698310	0.017955
Imidazoleacetic acid	0.795395	0.001849
Indole-3-acetamide	1.092299	0.036835
Tryptamine	-1.076970	0.004888
D-Proline	-3.192060	0.003220
L-Phenylalanine	0.914650	0.029405
L-Dopa	0.712119	0.027122
L-Tyrosine	0.752152	0.002090
L-Threonine	0.866922	0.021382
Methylimidazoleacetic acid	1.172759	3.29E-07
p-Coumaric acid	1.029298	0.045943
Histamine	-1.484310	0.032054

## Discussion

Rhizome expansion is an important characteristic of Kentucky bluegrass, however, the factors affecting this characteristic has not been well understood [9, 22]. This study investigated the rhizome growth status of three Kentucky bluegrass germplasms with different phenotypes and its relationship with N and C in rhizome. To our knowledge, this is the first report of the connection between rhizome expansion and carbon and nitrogen metabolisms in Kentucky bluegrass. The results of this work indicate that carbon and nitrogen metabolisms in the rhizome belong to the core mechanisms relating to rhizome expansion capacity for Kentucky bluegrass. In this work, greater rhizome contents of carbohydrate and nitrogen compounds were found for the YZ germplasms, which has the strongest expansion among all three germplasms (Fig. 1). This indicated that carbon and nitrogen were important for the rhizome development of Kentucky bluegrass.

Rhizome is a form shoot branching. The structural features of rhizomes are similar to those of tillers [12]. Development and growth of rhizomes require energy and material, this should be the reason why the germplasms with the greatest expansion (YZ) had greater carbohydrate contents in its rhizomes. Similar results were found in many other plant species, such as garden pea [35], rice [36]. Kentucky bluegrass is a cool season turf grass. In October, the aboveground parts have largely stopped growing and energy begins to accumulate in the underground rhizomes for rhizome expansion and plant overwintering. Soluble sugar is directly involved in cell metabolism and other activities [37], and distributed in active tissues to provide energy [38]. Starch degrades to generate soluble sugars, whose content increases [39]. Starch is the major storage form of carbohydrates and an optimal carbon source in plants [40]. Sucrose acts as the end product of photosynthesis [41], and plays an important role in the shoot branching [42]. In the *moc* 2 mutant of rice, fructose-1,6-bisphosphatase was disrupted in the stem, which leads to the insufficient sucrose, thereby contributing to a decrease tiller number [16]. Sucrose promotes shoot outward growth in a dose-dependent manner by inhibiting the expression of D3 protein and alleviating the degradation of D53 [41], which may lead to enhanced rhizome expansion. This is similar to a previous study which suggested that sucrose could promote the horizontal growth of rhizome buds of *Oryza longistaminata* [43]. Cai et al. [44] demonstrated that tobacco (*Nicotiana tabacum*) transformants increase growth rate by increasing sucrose transporter proteins in sucrose medium. Spaying with sucrose increased the sucrose content and enzyme (SPS and SS) activity of apple plants, which keeps the N metabolism in balance and promotes plant growth [45]. SPS is a key enzyme that promotes sucrose synthesis. In the present work, similar version was observed for sucrose and SPS, with maximum values in the YZ (Table 1). More recently, Wang et al. [17] also demonstrated that sucrose can affect the bud growth via glycolysis and OPPP in rose. Sucrose also acts as a signaling molecule and stimulates the growth of buds in plant [46–48], this may be the case of Kentucky bluegrass in this work.

Regulations of carbon and nitrogen metabolism in plants are mutually coupled and restricted [23]. Different cultivars of Kentucky bluegrasses possess different N uptake and assimilation [22]. The present work found that the Kentucky bluegrass with the strongest expansion capacity had the highest inorganic nitrogen in its rhizome (Fig. 3). This may imply that the inorganic nitrogen in the rhizome is important for rhizome development and plant growth for Kentucky bluegrass expansion. Similar to the findings of the present work, Shibasaki et al. [12] found a positive correlation between plant nitrogen content and its expansion capacity. Plant roots can absorb inorganic nitrogen from soil. Rhizome is the main organ for the horizontal expansion of Kentucky bluegrass, and can produce roots at the nodes [7]. Adequate nitrogen contributes to nutritional reproduction, promotes horizontal field expansion, and shapes plant growth and development [22]. The resulting altered rhizome architecture may further affect relationship of rhizome - N. Hence, NO_3_^−^-N might facilitate the growth and development of rhizome, by acting not only as a main form of inorganic nitrogen but also perhaps as a signaling molecule [49, 50]. Guo et al. [51] used the ^15^ N isotope labeling method, finding that much nitrogen accumulates in the rhizome, which helps it expand across the belowground space. In the present study, GS and NR activity showed the same trend as NO_3_^−^-N content, although differences in NR activity were not significant (Table 1). The key to plant growth is the conversion of inorganic nitrogen into amino acids, and GS plays an appreciable role in this process [52, 53]. As demonstrated by Shibasaki et al. [12], inorganic nitrogen can quickly promote the synthesis of glutamine, thereby increasing the content of cytokinin, and promoting the formation and growth of rhizome axillary buds, similarly to our results.

**Fig. 7:**
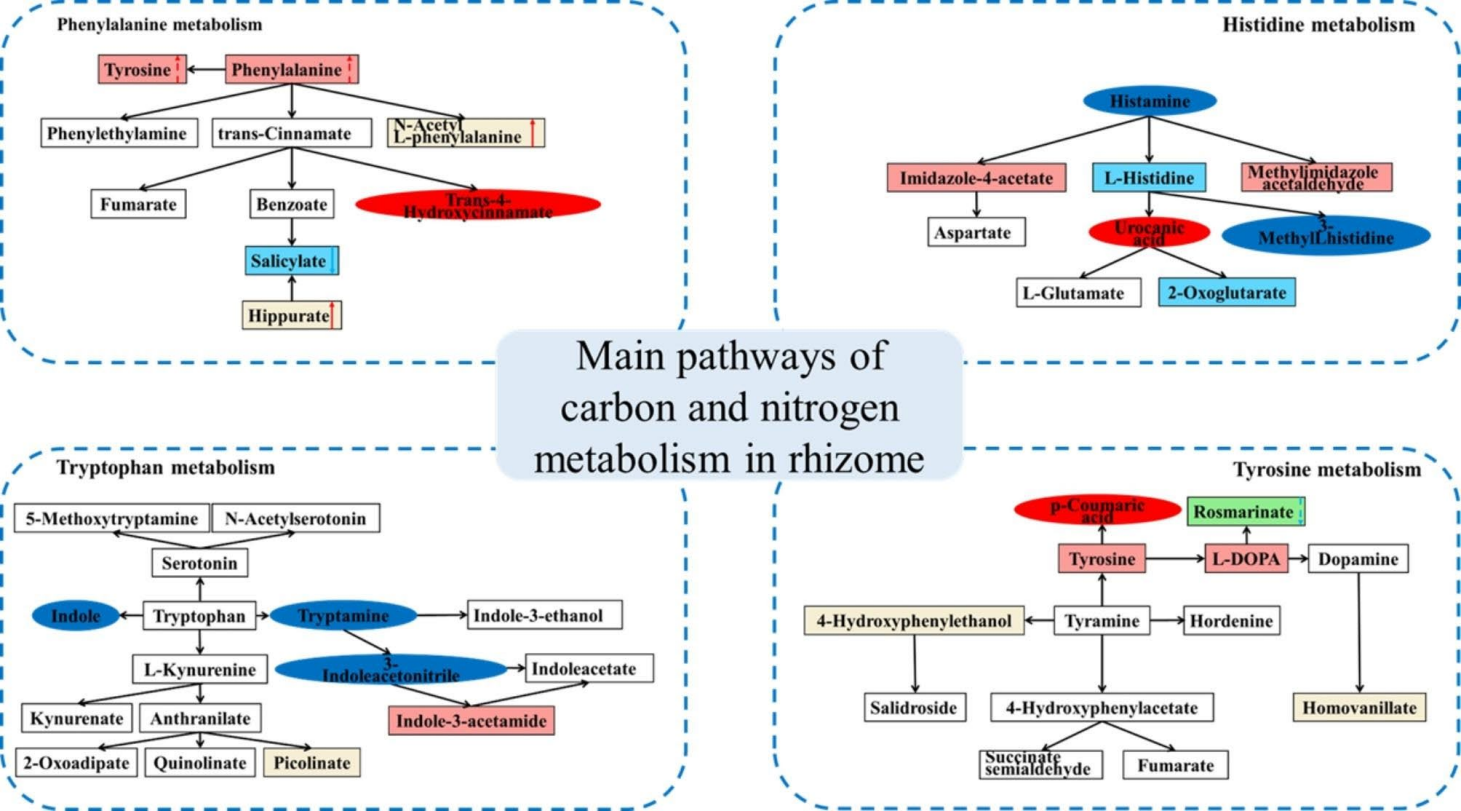
Main pathways of carbon and nitrogen metabolism in rhizome. Boxes represent annotated metabolites by the corresponding pathway, the blue ellipse represent metabolites of AD and WY downregulated relative to YZ, the red ellipse represent metabolites of AD and WY upregulated relative to YZ, the light blue boxes represent metabolites of AD downregulated relative to YZ, the light yellow boxes represent metabolites of AD upregulated relative to YZ, the light red boxes represent metabolites of WY downregulated relative to YZ, the light green boxes represent metabolites of WY upregulated relative to YZ

Amino acids not only provide nitrogen nutrients for plants but also participate in energy metabolism in the plant as a source of carbon. Therefore, adequate amino acid supply is important for promoting rhizome growth and development. High amino acid content in rice leaves is beneficial for tiller formation and growth [21]. Rhizome growth of Kentucky bluegrass is correlated with amino acid metabolism [7]. Amino acid metabolism networks show cross-regulation [54]. We used LC-MS to identify four metabolic pathways with carbon and nitrogen metabolism (Fig. 5). Interestingly, in the present study, all four metabolic pathways are aromatic amino acids, which have a high degree of biosynthesis consistency. Among them, tryptophan metabolism is the main pathway for plants to synthesize auxin. Auxin can regulate cell division, elongation, and differentiation, thereby regulating the phenotypic plasticity of plants, and promoting their branching [55, 56]. Ma et al. [9] found that the regenerated of new rhizomes was accompanied by the increase in auxin content and genes. We used PLS-DA and KEGG pathway analysis and screened out eight metabolites (Fig. 6). Our results showed that indole, 3-indoleacetonitrile, and tryptamine contents were significantly higher in the YZ than AD and WY in tryptophan metabolism (Figs. 6 and 7). Indole is the precursors of the Trp-independent IAA biosynthesis, and 3-indoleacetonitrile and tryptamine are important part of the Trp-dependent pathways [57]. Plants influence the morphological characteristics of rhizome development by regulating the enrichment of auxin signals in the rhizome [26]. Previous studies have also shown that the biosynthesis of local auxin is a key role in the growth and development of plant organs [58]. The regular supply of maternal auxin ensured the complete development of embryos in *Arabidopsis* [59]. Rhizome buds and tiller buds originated from the stem base. Interestingly, the increase in indole-3-acetic acid (IAA) transport was synchronized with tiller development [60]. Auxin is also affected by phenylalanine metabolism [61]. There is a metabolic crosstalk between the biosynthesis of cytoplasmic phenylalanine and that of tryptophan-dependent auxin, this mediated by aminotransferase and phenylpyruvate (as the amino receptor in the synthesis), to increase the content of petunia auxin. We found that differences in the phenylalanine metabolic pathway existed in the rhizome of different Kentucky bluegrass materials. As a precursor to many plant compounds, phenylalanine connects the primary and secondary metabolism of plants. Plants transfer large amounts of carbon to lignin via phenylalanine metabolism [62], and Ito et al. [63] confirmed that the rhizome of moso bamboo is highly lignified. In this study, NH_4_^+^-N contents in rhizomes were not significantly different, perhaps because the rhizome of Kentucky bluegrass mainly absorbs NO_3_^−^-N through mass flow [64].

## Conclusion

Overall, noticeable variation was present across the rhizomes of different Kentucky bluegrass materials. The Kentucky bluegrass with strong rhizome expansion exhibited the highest rhizome number and tiller number and the greatest carbon (soluble sugar, starch, sucrose, sucrose phosphate synthase) and nitrogen metabolism (NO_3_^−^-N, free amino acid, glutamate dehydrogenase and glutamine synthetase). Furthermore, amino acid and carbohydrate metabolites (indole, 3-methylhistidine, tryptamine, 3-indoleacetonitrile, and histamine) exhibited an enhanced accumulation in strong rhizome expansion. Tryptophan metabolism and phenylalanine metabolism may be the main metabolic pathway for enhancing rhizome expansion. These results reveal pronounced differences in carbon and nitrogen metabolism in the rhizomes of different materials, which can provide new insights for the molecular breeding of rhizomatous plants.

## Materials and methods

### Kentucky bluegrass germplasms

In our previous work, based on rhizome plant number, rhizome volume, rhizome surface area, aboveground plant volume, stem length, tiller number, and largest rhizome extension distance that had the different expansion capacity wild Kentucky bluegrass materials were screened out [65]. Three Kentucky bluegrass germplasms were used in this work, they have strong, medium, and weak rhizome expansion capacity germplasms and were named Yuzhong (YZ), Weiyuan (WY), and Anding (AD), respectively (Additional file 1: Table S1). The seeds of these Kentucky bluegrass were collected from distinctly different habitats in Gansu Province and maintained in the germplasm collections of Gansu Agricultural University, China. A detailed list of plant material is reported in Chen [65]. The use of this material complies with the relevant agency or national legislation and guidelines.

### Field experiment

The field work was carried out in the experimental farm of Gansu Agricultural University in Lanzhou City, Gansu Province, China (36°48′N, 103°3′E, Altitude 1517 m). Lanzhou has a temperate continental climate, with a mean annual temperature of 10 °C, and a mean annual rainfall of 362 mm. The soil in the farm belongs to loess loam. In the 0–40 cm soil layer, the organic matter, alkali-hydrolyzable nitrogen, available phosphorus and available potassium were 2.34%, 40.37 mg·kg-1, 45.62 mg·kg-1 and 155.68 mg·kg-1, respectively. The pH was 7.5.

Each Kentucky bluegrass had 12 plots in the field work, thus there were 36 plots (each 0.5 × 0.5 m) with a spacing of 1 × 1 m. In June 2019, Kentucky bluegrass seeds were sterilized and randomly assigned to the plots. Twenty-one days after germination, plants in each plot were thinned to 12. Plants grew under natural sunlight and temperature. The plants were harvested in October 2020, with all the plants in each plot harvested for analysis. Whole plants were separated into rhizome and aboveground and rinsed sequentially with tap water and deionized water for three times. A portion of these rhizome samples were kept for morphology measurements and the remaining samples used for physiological parameter and metabolite determinations (stored at -80 °C). The metabolomics analysis was conducted with six replicates. The other measurements in this study were three replicates.

### Morphology measurement

The numbers of tillers and rhizomes of each plant were counted, dry weights were determined after the samples being dried at 80 °C for 48 h. The dried samples were used for subsequent analysis.

### Nitrogen analysis

Fresh plant samples were extracted in boiling water bath, then the extractant NO_3_^−^ content was measured with salicylic acid colorimetry test according to Cataldo et al. [66], NH_4_^+^ content was determined by the Berthelot color reaction method according to Gordon et al. [67], while free amino acids content was determined by the ninhydrin method [68] (UV-vis spectrophotometer, TU-1901, China).

### Carbohydrate analysis

Dry rhizome samples were extracted with ethanol at 80℃, then the extractant was used for soluble sugar [69] and sucrose analysis [70]. The extraction residue of soluble sugar was collected and extracted in boiling water. The starch content was assayed using the anthrone reagent [69] (*UV*-vis spectrophotometer, TU-1901, China).

### Rhizome enzyme activity analysis

Frozen rhizome samples were used for enzyme activity analysis. Sucrose synthase (SS) and sucrose phosphate synthase (SPS) activities were determined according to the method of Sicher et al. [71]. Nitrate reductase (NR) activity was determined according to the method of Cambraia et al. [72]. Glutamine synthetase (GS) activity was determined according to the method of Oaks et al. [73]. Glutamate synthase (GOGAT) activity was determined according to the method of Kok et al. [74]. Glutamate dehydrogenase (GDH) activity was determined according to the method of Cren et al. [75]. Acid invertase (AI) and Neutral invertase (NI) activity was determined according to the method of Liu et al. [76].

### Metabolic profiling

Untargeted metabolomics was performed using liquid chromatography-mass spectrometry (LC-MS). Rhizome sample preparation and sequencing were performed at Novogene (Beijing, China). Briefly, 100 mg of each rhizome sample was first ground with liquid nitrogen and then homogenate added to a 500-µl aqueous solution of 80% methanol. After centrifugation, the ensuing supernatant was collected and injected into the LC-MS system for analysis [77].

Samples were injected onto a Hypesil Gold column (100 × 2.1 mm, 1.9 μm), using a 17-min linear gradient at a flow rate of 0.2 mLmin^− 1^. The mobile phase of the positive polarity mode was eluent A (0.1% FA in Water) and eluent B (Methanol). The mobile phase of the negative polarity mode was eluent A (5 mM ammonium acetate, pH 9.0) and eluent B (methanol). The operating solvent gradient was set as follows: 2% B, 1.5 min; 2–100% B, 12.0 min; 100% B, 14.0 min; 100–2% B, 14.1 min; 2% B, 17 min. The spray voltage was 3.2 kV, the capillary temperature was 320 °C, the sheath gas flow rate was 40 arb, and the aux gas flow rate was 10 arb.

Raw files were processed in Compound Discoverer 3.1 software (CD 3.1, ThermoFisher), which performed the peak alignment, peak picking, and quantitation for each metabolite. Next, the molecular formulas were aligned to the mzCloud (https://www.mzcloud.org/), mzVaul, and Masslist database. Statistical analyses were performed using R v4.0.2 software.

The detected metabolites were annotated using the KEGG (https://www.genome.jp/kegg/pathway.html) [78, 79], HMDB (https://hmdb.ca/ metabolites), and LIPID Maps (http://www.lipidmaps.org) databases. A partial least squares discriminant analysis (PLS-DA) of the identified metabolites implemented using was metaX [80]. We applied univariate analysis (Student t-test) to calculate the statistical significance of each metabolite content between the two groups significance (P-value). The criterion for identified metabolites was variable importance in the projection (VIP) > 1 and *P*-value < 0.05.

### Statistical analysis

Morphological and physiological data of different Kentucky bluegrass were processed with SPSS (SPSS Inc., Chicago, IL, USA) using one-way ANOVA with Tukey’s test, the graphs were generated with Origin Pro (version 9, Origin Lab). The metabolomics analysis was run in R Software (version 4.0.2, R Foundation, Vienna, Austria). Significance was set at *P* < 0.05.

## Electronic supplementary material

Below is the link to the electronic supplementary material.

Supplementary Material 1

## Data Availability

The raw data in the study are available on further inquiries from the corresponding author.

## Abbreviations

GS: Glutamine synthetase
GDH: Glutamate dehydrogenase
GOGAT: Glutamate synthase
NR: Nitrate reductase
SS: Sucrose synthase
SPS: Sucrose phosphate synthase
NI: Neutral invertase
AI: Acid invertase
YZ: *Poa pratensis* (Yuzhong)
WY: *Poa pratensis* (Weiyuan)
AD: *Poa pratensis* (Anding)
DEMs: Differentially expressed metabolites
KEGG: Kyoto Encyclopedia of Genes and Genomes
LC-MS: Liquid chromatography coupled to mass spectrometry
OPPP: Oxidative pentose phosphate pathway
PLS-DA: Partial least squares discriminant analysis
VIP: Variable importance in the projection
IAA: Indole-3-acetic acid
PCA: Principal component analysis
