# Supplementary material for: Carbon and nitrogen metabolism affects kentucky bluegrass rhizome expansion
Source: BMC Plant Biol. 2023 Apr 26;23:221. doi: 10.1186/s12870-023-04230-x (PMC10131326; doi:10.1186/s12870-023-04230-x)
Supplement: Supplementary file 1 — Supplementary Material 1 [file 12870_2023_4230_MOESM1_ESM.docx]

**Additional file 1: Table S1.** Original habitats of the three Kentucky bluegrass plants used in this work

| **Code** | **Material name** | **Collection site** | **Altitude (m)** | **Geographical coordinate** | **Habitat** | **Rhizome expansion** |
| --- | --- | --- | --- | --- | --- | --- |
| YZ | *Poa pratensis* (Yuzhong) | Yuzhong, Gansu | 1965 | 35°48 N  104°04 E | Ditch | Strong |
| WY | *Poa pratensis* (Weiyuan) | Weiyuan, Gansu | 2401 | 35°02 N  104°05 E | Roadside | Medium |
| AD | *Poa pratensis* (Anding) | Anding, Gansu | 2035 | 35°58 N  104°62E | Hillside | Weak |

**Additional file 2: Table S2.** Four common KEGG pathways and related metabolites in AD vs YZ and WY vs YZ.

| **Pathway name** | **Number of total metabolites** | **Number of differential metabolites** | |
| --- | --- | --- | --- |
|  |  | **AD vs YZ** | **WY vs YZ** |
| Histidine metabolism | 9 | 6 | 5 |
| Tyrosine metabolism | 14 | 3 | 4 |
| Tryptophan metabolism | 16 | 4 | 4 |
| Phenylalanine metabolism | 14 | 4 | 3 |


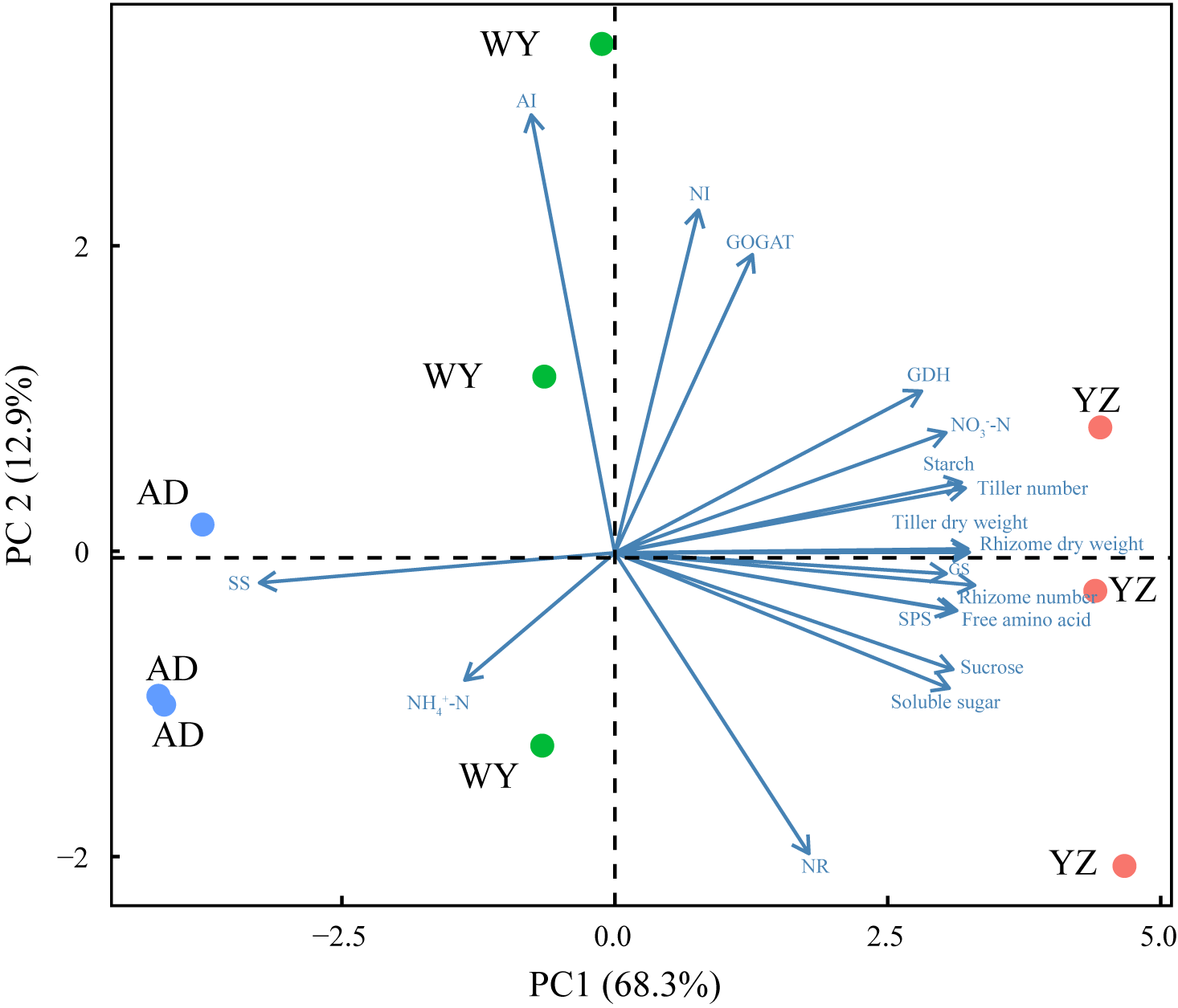


**Additional file 3: Figure S1.** Scores plot of the principal component analysis. PC1, first principal component; PC2, second principal component. A red dot represents the YZ plant, a green dot represents the WY plant, and a blue dot represents the AD plant.


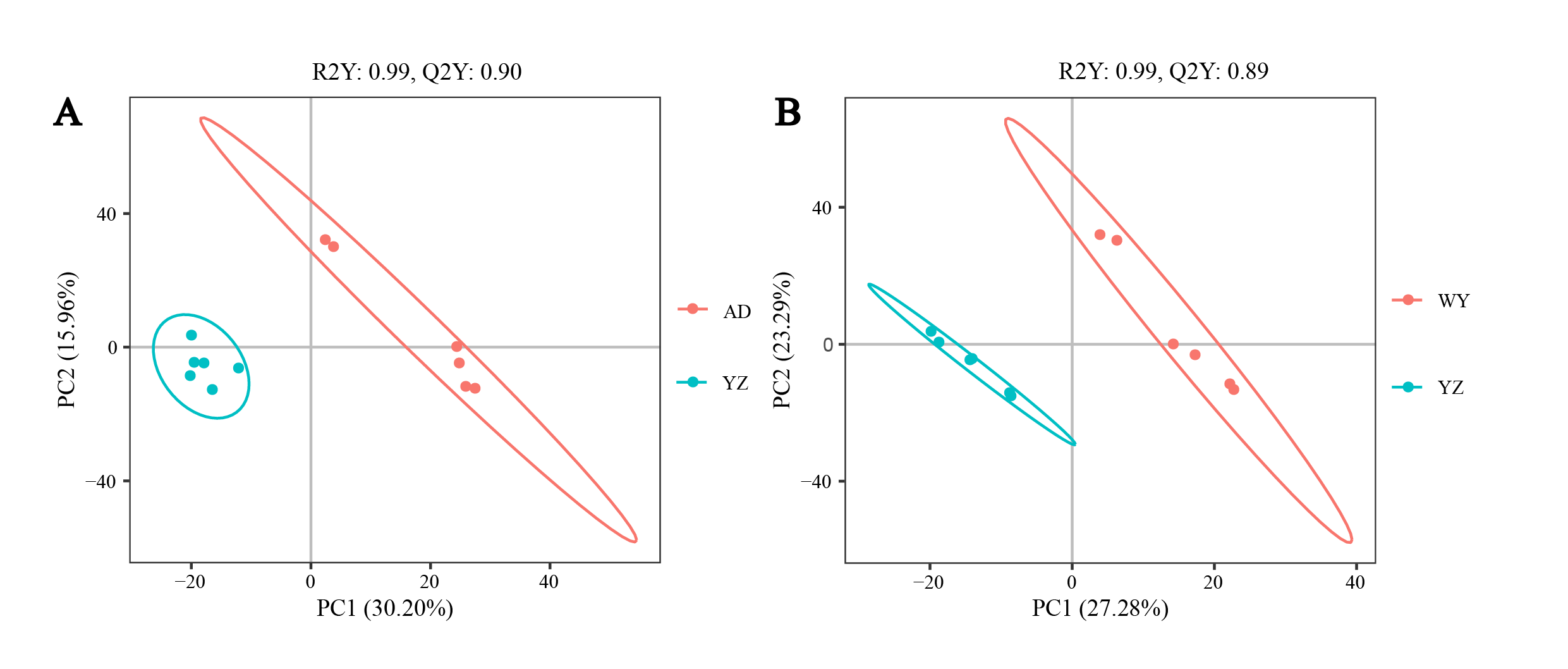


**Additional file 4: Figure S2.** PLS-DA of metabolite profiles of different rhizomes in the AD vs. YZ (A) and the WY vs. YZ (B). Each dot represents an individual sample.


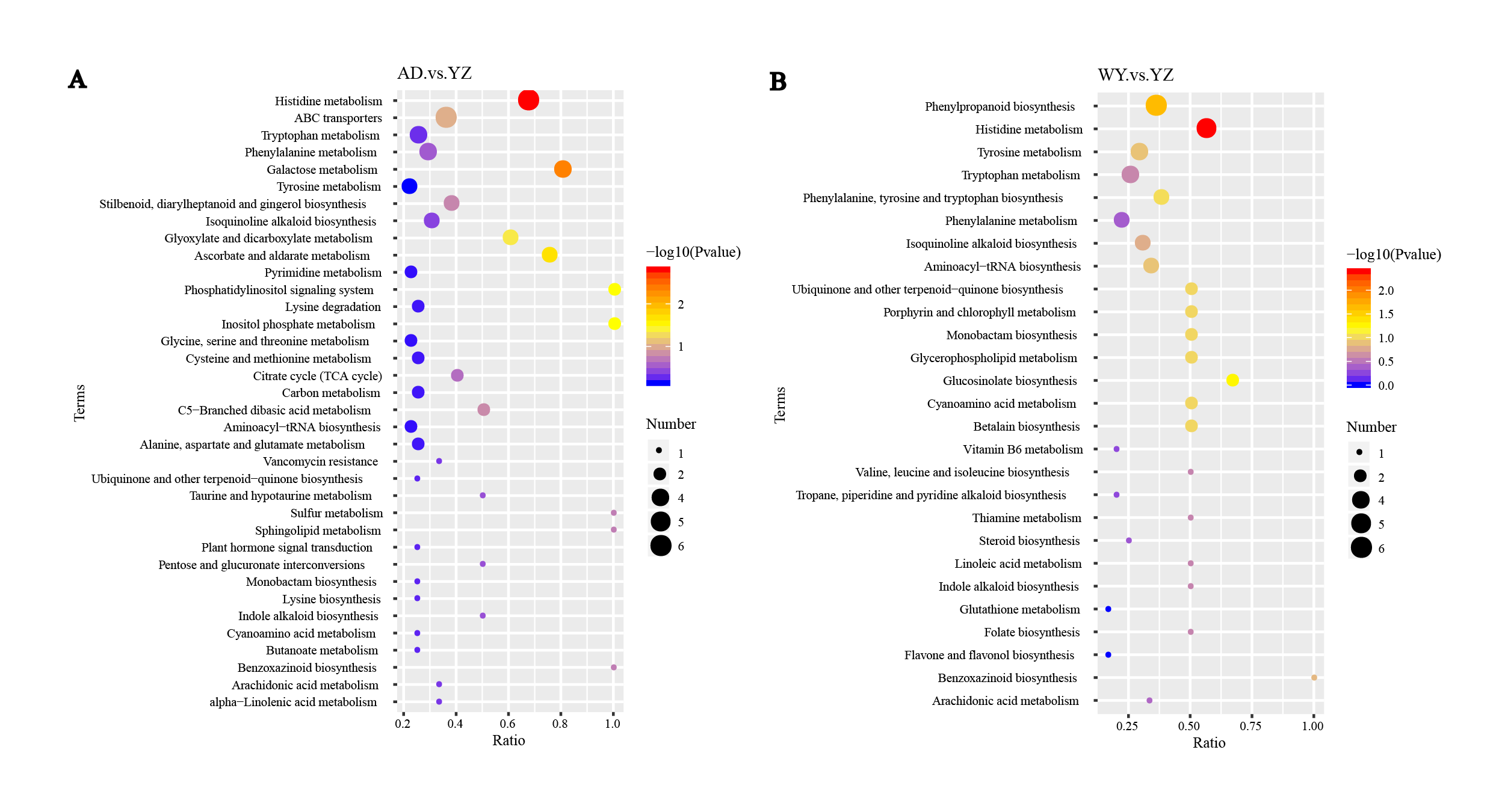


**Additional file 5: Figure S3.** KEGG pathways of DEMs in AD vs YZ (A) and WY vs YZ (B). The size of the circle represents the number of metabolites.
